# Registration of clinical trials: An idea whose time has come!

**DOI:** 10.4103/0253-7613.41037

**Published:** 2008

**Authors:** Arun Bhatt

**Affiliations:** ClinInvent Research Pvt Ltd, A-103, Everest Chambers, Andheri-Kurla Road, Marol, Andheri (East), Mumbai - 400 059, India. E-mail: arunbhatt@clininvent.com

In recent years, registration of clinical trials has progressed from being desirable and voluntary to being essential and mandatory. This is mainly due to the editorial activism of leading medical journals and the vigilance of academic researchers.[1–4] When we reviewed the global status of clinical trial registration in 2006,[5] the impact on the pharmaceutical sponsors and research investigators of the International Committee of Medical Journal Editors' (ICMJE) 2005 initiative was just beginning to be felt. In 2005, ICMJE recommended that its member journals demand, as a precondition for consideration for publication, registration in a public trials registry.[2] The ICMJE policy initiative aimed to ensure that information about the existence and design of any clinical trial was publicly available so as to curb the practice of selective reporting of clinical trials.[2] Initially, the pharmaceutical industry and the researchers were resistant to these initiatives, fearing that registration would be burdensome and would stifle competition.[6] However, the response has since been overwhelming. Before the ICMJE policy initiative, ClinicalTrials.gov contained 13,153 trials; this number jumped to 22,174 within a month.[3] In April 2007, the registry contained over 40,000 trials, with more than 200 new trials being added weekly.[6] The four other registries have also grown as more and more journals adopted the ICMJE clinical trials registration policy.

The ICMJE policy has been supported by other governmental and non-governmental efforts. The US Food and Drug Administration (FDA) Revitalization Act, which came into force on September 27, 2007, aims to improve the FDA's ability to ensure the safety of drugs and medical devices. This new Act includes a specific provision on clinical trial registration;[7] the act requires all sponsors of clinically directive therapeutic trials to register their studies, at inception, in a public database sponsored by the National Library of Medicine.[7] The World Health Organization's (WHO) International Clinical Trial Registry Platform (ICTRP), which was in a nascent stage in 2005, has matured and now provides a wide array of registries.[6] The ICTRP has taken the first steps toward developing a network of primary and partner registers that meet WHO-specified criteria.[8] The primary registers are WHO-selected ones managed by not-for-profit entities that will accept registrations for any interventional trials, delete duplicate entries from their own register, and provide data directly to the WHO. The Indian registry, Clinical Trials Registry-India (CTRI), is one of the WHO-ICTRP compliant primary registries.[8,9] The partner registers will include those that submit data to the primary registers but limit their own register to trials in a restricted area (such as a specific disease, company, academic institution, or geographic region).[8] The WHO's efforts, through the ICTRP, to develop a coordinated process for identifying, gathering, de-duplicating, and searching trials from registries around the world will eventually provide a one-stop search portal for those seeking information about clinical trials.[6]

The Indian policy framework for registering clinical trials has been evolving over the last 2 years.[5] In our 2006 editorial, we had discussed the need to develop two separate registries: one regulatory clinical trial registry (RCTR) and another academic clinical trial registry (ACTR). We had also supported Dr. Gitanjali's plea to the editors of Indian medical journals to endorse the ICMJE statement.[5] The July 2007 launch of CTRI[9] and the 2008 statement on the publishing of clinical trials[10] from editors of 12 leading Indian journals are noteworthy Indian efforts in support of the ICMJE and WHO initiatives.

The CTRI is hosted at the National Institute of Medical Statistics (NIMS), with funding from the Department of Science and Technology, Indian Council of Medical Research (ICMR), and the WHO. It is a free and searchable online register of all clinical trials being conducted in India.[9] The CTRI is based on WHO's guidelines and is compatible with ICTRP.[8] This registry requires all interventional clinical trials conducted in India and involving Indian participants to be registered. It will also accept clinical trial registration from researchers of other countries that do not have the facilities for such registration.[9] The CTRI adopts the WHO definition of a clinical trial: ‘any research study that prospectively assigns human participants or groups of humans to one or more health-related interventions to evaluate the effects on health outcomes.’[8,9] Health-related interventions include any intervention used to modify a biomedical or health-related outcome (for example, drugs, surgical procedures, devices, behavioural treatments, dietary interventions, and process-of-care changes). Health outcomes include any biomedical or health-related measurements obtained in patients or participants, including pharmacokinetic measurements and adverse events.[6,9]

For registration of a trial, the CTRI requires the following information as per WHO-ICTRP:[8,9] (1) unique trial identification number (UTRN), (2) date of registration in primary register, (3) secondary identification number(s), (4) source(s) of monetary or material support, (5) primary sponsor, (6) secondary sponsor, (7) contact for public queries, (8) contact for scientific queries, (9) public title, (10) scientific title, (11) countries of recruitment, (12) health condition(s), (13) interventions, (14) key inclusion and exclusion criteria, (15) study type, (16) date of first enrolment, (17) target sample size, (18) recruitment status, (19) primary outcome(s), and (20) key secondary outcomes. In addition, the CTRI also requires declaration of few a additional facts[9] at the time of registration; these are: (1) principal investigator's name and address, (2) name of the ethics committee and approval status, (3) regulatory clearance obtained from DCGI, (4) estimated duration of trial, (5) site/s of study, (6) phase of trial, (7) brief summary, (8) method of generating randomization sequence, (9) method of allocation concealment, and (10) blinding and masking.

The ‘responsible registrant’ for a trial, either the principal investigator (PI) or the primary sponsor, should register the trial before the enrolment of the first patient. The primary sponsor is ultimately accountable for ensuring that the trial is properly registered. For multi-center and multi-sponsor trials, the lead PI or the lead sponsor should take responsibility for registration. However, in case of multi-country trials, the Indian PI should also get the trial registered in CTRI, quoting any other registration number as its secondary ID.[9] At present, trial registration in CTRI is voluntary.

After its launch, the CTRI along with the Indian Journal of Medical Research (IJMR) organized a meeting of the editors of Indian biomedical journals to evolve a policy to be followed for publication of clinical trials in Indian biomedical journals. The meeting, attended by editors of 12 Indian biomedical journals, unanimously decided that the editors have the responsibility to promote the registration of all clinical trials being conducted in India and they must urge researchers to register their trials within the stipulated time so as to make the clinical trial data transparent and to enable the results to be published in good journals.[10] Subsequently, the editors of these journals published a statement urging all those conducting and/or planning to conduct clinical trials involving human subjects to register their trials in CTRI or in any primary clinical trial register.[10] From January 2010 onwards, these journals will consider publication of a trial (started in or after June 2008) only if it has been registered prospectively.[10]

The CTRI has provided an Indian option for Indian investigators and Indian pharmaceutical sponsors. The response has been overwhelming: within 3 months of its launch over 90 clinical trials have already been registered.[10] However, CTRI is unlikely to get a similar response from global sponsors who have been registering their trials in ClinicalTrials.gov. This directory contains 334 India based trials, most of which are international trials being carried out at Indian sites.[11] Some Indian sponsors have also registered their trials in ClinicalTrials.gov. The need for the declaration of some additional items for registration in CTRI will also make sponsors and investigators seek registries which are less demanding. The CTRI and its sponsors need to take active steps to sensitize investigators, funding agencies, ethics committee members, pharmaceutical companies, health professionals, and medical journal editors on the need to register all clinical trials.

The efforts of ICMJE and WHO have made registration of clinical trials the norm across the world. The next logical step would be the registration of trial results.[6,7] The ICMJE has already recommended a standard abstract format for reporting of results and suggested that parties interested in results registration should consider requiring the deposition of such an abstract in the registry 24 months after closure of data collection if the results have not been published in a peer-reviewed venue by that time.[6] When submitting a paper, authors should fully disclose to editors all posting in registries of results of the same or closely related work. There has also been a call to the trial subjects to take part only in those studies whose sponsors have fully registered them in an appropriate public database and agreed to publish the results of the study.[7] The WHO and ICMJE's global efforts, coupled with legislative changes, will lead to fully open clinical trials, ie, trials which are accessible to public. Ultimately, this should lead to more effective and safer treatment for patients.[6,7] Registration of clinical trials will facilitate the dissemination of information among clinicians, researchers, and patients. It will also help in assuring trial participants that the information that accrues as a result of their altruism will become part of the public record.[6] The international efforts to promote registration of clinical trials will go a long way in increasing public trust and confidence in clinical research and medical science.

## References

[1] De Angelis C, Drazen JM, Frizelle FA, Haug C, Hoey J, Horton R (2004). Clinical trial registration: A statement from the International Committee of Medical Journal Editors. N Engl J Med.

[2] De Angelis C, Drazen JM, Frizelle FA, Haug C, Hoey J, Horton R (2005). Is this clinical trial fully registered?. A statement from the International Committee of Medical Journal Editors. N Engl J Med.

[3] Zarin DA, Tse T, Ide NC (2005). Trial registration at ClinicalTrials.gov between May and October 2005. N Engl J Med.

[4] Drazen JM, Wood AJ (2005). Trial registration report card. N Engl J Med.

[5] Bhatt A, Sewlikar S (2006). Registering clinical trials in India. Natl Med J India.

[6] Laine C, Horton R, DeAngelis CD, Drazen JM, Frizelle FA, Godlee F (2007). Clinical trial registration-looking back and moving ahead. N Engl J Med.

[7] Drazen JM, Morrissey S, Curfman GD (2007). Open clinical trials. N Engl J Med.

[8] World Health Organization International Clinical Trial Registry Platform http://www.who.int/ictrp/about/details/en/index.html.

[9] Clinical Trials Registry - India, National Institute of Medical Statistics (ICMR) http://www.ctri.in/clinicaltrials/rials_jsp/index.jsp.

[10] Satyanarayana K, Sharma A, Parikh P, Vijayan VK, Sahu DK, Nayak BK (2008). Statement on publishing clinical trials in Indian biomedical journals. Indian J Ophthalmol.

[11] http://www.ClinicalTrials.Gov.

